# Mechanosensitive channels: feeling tension in a world under pressure

**DOI:** 10.3389/fpls.2014.00558

**Published:** 2014-10-21

**Authors:** Rémi Peyronnet, Daniel Tran, Tiffanie Girault, Jean-Marie Frachisse

**Affiliations:** ^1^National Heart and Lung Institute, Imperial College LondonLondon, UK; ^2^Institut des Sciences du Végétal – Centre National de la Recherche Scientifique, Saclay Plant SciencesGif-sur-Yvette, France

**Keywords:** stretch-activated channels, mechanotransduction, mechanobiology, cytoskeleton, plant, MSL, MscS, membrane tension

## Abstract

Plants, like other organisms, are facing multiple mechanical constraints generated both in their tissues and by the surrounding environments. They need to sense and adapt to these forces throughout their lifetimes. To do so, different mechanisms devoted to force transduction have emerged. Here we focus on fascinating proteins: the mechanosensitive (MS) channels. Mechanosensing in plants has been described for centuries but the molecular identification of MS channels occurred only recently. This review is aimed at plant biologists and plant biomechanists who want to be introduced to MS channel identity, how they work and what they might do *in planta*? In this review, electrophysiological properties, regulations, and functions of well-characterized MS channels belonging to bacteria and animals are compared with those of plants. Common and specific properties are discussed. We deduce which tools and concepts from animal and bacterial fields could be helpful for improving our understanding of plant mechanotransduction. MS channels embedded in their plasma membrane are sandwiched between the cell wall and the cytoskeleton. The consequences of this peculiar situation are analyzed and discussed. We also stress how important it is to probe mechanical forces at cellular and subcellular levels *in planta* in order to reveal the intimate relationship linking the membrane with MS channel activity. Finally we will propose new tracks to help to reveal their physiological functions at tissue and plant levels.

## INTRODUCTION

All organisms, from bacteria to mammals and plants, experience mechanical forces. These forces are ubiquitous and very diverse coming from both the internal and the external environment. One of the most common external sources of stimulation sensed by both plants and animals is touch (pressure, shear stress). Like animals, plants are sensitive to gravity which guides their growth with respect to the gravity vector. Cells also generate their own intracellular forces as is obvious during cell division, cell elongation, or adjustment after osmotic challenge. While animals have to deal with circulating liquids (blood and urinary) and gases (lungs) as well as contractile elements (muscles), plant cells with their high turgor pressure represent very peculiar and interesting living systems from a mechanical point of view.

**FIGURE 1:**
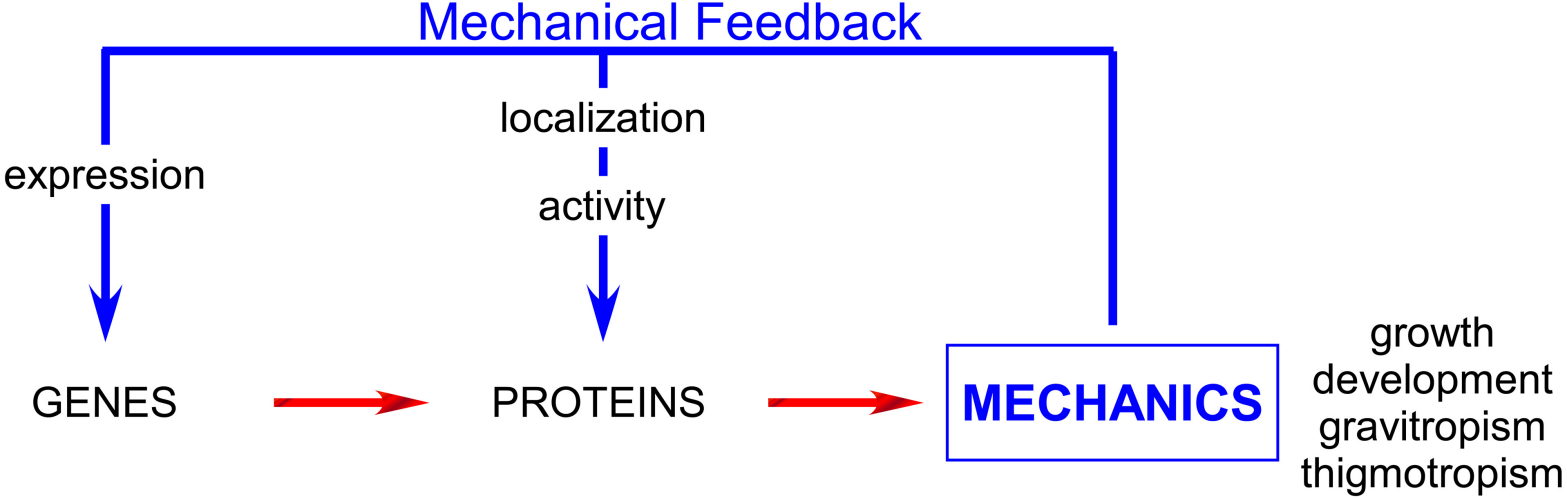
**Mechanical feedback affects the whole plant physiology acting on both gene and proteins and plays major role especially in development, growth and tropisms**.

Over the last few years, it has become apparent that the ability of cells to sense and adapt to these forces is crucial for a wide range of biological processes. After two decades, during which the vast majority of studies were devoted to the dissection of gene regulatory pathways, mechanics is now being progressively integrated into the network, both as output (the impact of genes on cell mechanics) and input (the impact of mechanical signals on gene activity; **Figure 1**). Emerging techniques and tools now enable the measurement and manipulation of mechanical forces *in vitro* and progressively *in vivo.* This has led to an ongoing renaissance in studying mechanics.

Cells not only survive mechanical stimulation but also use it as a driving force to design their own architecture and to serve biological functions. This explains why cells and organisms have established mechanosensors. Three groups of proteins can fulfill this function: linkage proteins, structural elements, and MS ion channels.

Amongst the most well-known linkage proteins are integrins. These proteins are embedded in the plasma membrane and allow a mechanical coupling between the extra cellular matrix and the actin cytoskeleton. They are well described in the animal field and integrin-like proteins are reported in plants. NDR1 (Non-race-specific Disease Resistance1) for example is suggested to play a role in plasma membrane-cell wall adhesion and is required during plant-pathogen interaction (Knepper et al., 2011). In plants, serine-threonine protein kinases associated with the cell wall are good candidates to transduce mechanical forces. Among them are WAKs and THESEUS receptor-like kinase (RLKs; Monshausen and Gilroy, 2009) and other members of the large family of membrane-localized RLKs (Monshausen and Haswell, 2013). The cytoskeleton can be involved in several steps during mechanotransduction processes as demonstrated extensively in the animal field. It can directly transmit mechanical forces across the cell (Hayakawa et al., 2012) and also greatly control membrane tension (Gauthier et al., 2012) and organization (Jaqaman and Grinstein, 2012).

In this review we will focus on the most well-known mechanosensors: the MS channels.

## MS CHANNELS: A COMMON FEATURE OF LIVING ORGANISMS FROM BACTERIA TO MAMMALS

Mechanosensitive channels are fascinating proteins, being able to serve both as sensors and effectors. Embedded in membranes, they convert mechanical stimuli such as in-plane membrane tension and curvature into electrical or biochemical signals, leading to regulation of a wide repertoire of cellular processes allowing adaptive response. Directly gated by the mechanical stimulus, MS channels convert (within milliseconds) a mechanical force into electrical trans-membrane potential variation. Therefore MS channels are (with phototransduction) the most rapid transducers known to date in biological systems.

Mechanosensitive channels were discovered in embryonic chick skeletal myocytes by Guharay and Sachs (1984) using the patch-clamp technique (Hamill et al., 1981). Then, in 1987 the first recordings were obtained on bacteria (Martinac et al., 1987) and were followed one year later by the first recordings on plants (Falke et al., 1988). It was at this time that the hunt for molecular candidates began. The first MS channel to be cloned was the MscL (MS channel Large conductance) from *Escherichia coli* in 1994 (Sukharev et al., 1994b) and a second important step was the cloning of the first mammalian MS channel in 1998 (Patel et al., 1998). Since then, deciphering organism genomes has provided several protein candidates for mechanosensing. Numerous other MS channels have been identified but it is only recently (20 years after the first recording of a plant MS channel) that MS channels belonging to two different families were identified in plants (Nakagawa et al., 2007; Haswell et al., 2008). Mid1-Complementing Activity (MCA) exhibiting 10% identity to yeast Mid1 (Nakagawa et al., 2007) and correlated to Ca^2+^ influx for MCA1 together with MSL (MscS-Like), homologs of the weakly selective bacterial stretch-activated MscS channels, have opened a new field of investigation.

The combination of genomics, molecular, and electrophysiological approaches is starting to provide exciting information on the role of MS channels from mechanical perception to organism behavior.

In this review, rather than listing channel candidates characterized within several organisms, we will present some emblematic channels in bacterial and plant systems. We chose to present only well-electrophysiologically characterized channels whose membrane stretching represents a major regulation factor. Exhaustive overviews of the different MS channels characterized can be found notably in (Arnadottir and Chalfie, 2010; Martinac, 2011; Nilius and Honore, 2012; Kurusu et al., 2013; Monshausen and Haswell, 2013; Wilson et al., 2013).

### MscL AND MscS: THE BACTERIAL SAFETY-VALVES

Mechanical senses originated in unicellular organisms. In evolution, the first to appear was osmosensing, which allows a cell to maintain membrane integrity when confronted with varying aqueous environments. In fact, the most extensively characterized MS channels are the MscL (MS channel Large conductance) and MscS (Small conductance; Sukharev et al., 1994a; Levina et al., 1999) which were discovered 25 years ago in bacteria *E. coli* (Martinac et al., 1987) then cloned in 1994 and 1999 (Sukharev et al., 1994b; Levina et al., 1999) and crystallized in 1998 and 2002 (Chang et al., 1998; Bass et al., 2002). Both have very large conductances, or pore sizes, relative to eukaryotic channels, which are usually on the order of a few 10s of picosiemens; MscL, at about three nanosiemens, meaning a flux of 3 billion ions per second (at a membrane potential of 150 mV) is the largest gated channel, while MscS conductance is about one nanosiemens (**Table 1**). These two channels reflect distinct families of proteins. The MscS family, found in several species belonging to bacteria, archaea, algae, fungi, and plants, is quite diverse and a single organism may encode multiple members in its genome. For example, the genome of the model plant *Arabidopsis* encodes for 10 MscS-like (Haswell and Meyerowitz, 2006). The MscL channel is highly conserved, with only a single copy of the gene found in fungal and bacterial organisms. Unlike most of channels, Msc S and L have a lack of ionic specificity and are permeable to any charged molecule smaller than 1,000 molecular weight including proline, potassium glutamate, trehalose, and ATP (Hamill and Martinac, 2001). In respect to small ions, MscL is nonselective for both anions and cations (Martinac et al., 2008), whereas MscS exhibits a slight preference for chloride over potassium with a permeability ratio *P*Cl:*P*K in the range of 1.5–3 (Martinac et al., 2008). Each Msc (S or L) in *E. coli* exhibits a unique threshold tension for activation of ∼6.0 and ∼12.0 mN/m respectively, (Sukharev et al., 1999; Sukharev, 2002; Nomura et al., 2012). Biophysical approaches on MscS and MscL as well as studies performed on purified MscL, show that the protein alone reconstituted into liposomes retained mechanosensitivity, indicating that both channels directly sense membrane tension developed in the lipid bilayer alone (Sukharev et al., 2001; Perozo et al., 2002).

**Table 1:** Characteristics and functions of the four MS channels, MscL, MscS, TREK-1, and Piezo involved in mechanosensation in bacteria and mammals.

Channel	MscL	MscS	TREK-1	Piezo
Cloned from (organism)	*E. coli*	*E. coli*	*Mouse*	*Mouse*
Homologs in other organisms	Bacteria, archeabacteria, fungi	Bacteria, algae, fungi, archaebacteria, plant	Mammals	Mammals, plant, protozoa, invertebrates
Conductance	~3000 pS [a, b]	~ 1000 pS [a, b]	~50 pS [c]	~25–70 pS [d, n, k]
Selectivity	not-selective [b, e]	weak: Cl^-^ > K^+^ > metabolites [b, e]	K^+^ [c]	cation non-selective [d][f]
Activation	T_1/2_: ~12 mN.m^-1^ [g]	T_1/2_: ~6 mN.m^-1^ [h]	P_1/2_: -20 to -60 mm Hg [c, i, j]	P_1/2_: -25 to -48 mm Hg [d, k, l]
Inactivation	No	Yes (spheroplast) [a, m]	Yes, τ ~46 ms [c]	Yes, τ ~ 45 ms [n]
Activation factors	Membrane tension Membrane curvature [o]	Membrane tension Membrane curvature [p]	Membrane tension, Membrane curvature [q] heat, acdic pH, depol., ……	unknown
Functions	“Emergency release valve”	“Non-emergency release valve” Internal crowding sensor	Pain perception, ischemia, vasodilatation…	Red blood cell volume touch and pain perception …

*a: Perozo and Rees (2003), b: Sukharev et al. (1997), c: Honore et al. (2006), d: Coste et al. (2010):, e: Martinac et al. (2008), f: Bae etal. (2013), g: Sukharev et al. (1999), h: Sukharev (2002) i: Patel et al. (1998), j: Bang etal. (2000), k: Bae etal. (2011), l: Peyronnet et al. (2012), m: Sotomayor etal. (2007), n: Gottlieb etal. (2012), o: Mukherjee et al. (2014), p: Vasquez et al. (2008), q: Maingret et al. (2000).*

Bacteria are well documented for their ability to survive and grow in conditions of changing osmolarity. When they face a sudden hypoosmotic shock (which may take place, for instance, in gastrointestinal bacteria exposed to food processing, marine bacteria suddenly exposed to fresh water or soil bacteria trapped in rain water), a rapid influx of water will occur. Consequently, the mechanical membrane tension will rapidly rise. This should not exceed around 15 mN.m^-1^ (Evans and Ludwig, 2000; Boer et al., 2011). Above this level the rupture of the membrane will produce lysis of the cell. In order to avoid this situation, excessive membrane tension should be rapidly relieved. Based on experiments performed on knock-out (KO) bacteria, it was proposed by Levina et al. (Levina et al., 1999) that MscS and MscL represent two efficient “valves” acting synergistically allowing osmolyte eﬄux after swelling. MscS is the non-emergency “valve” while MscL represents the ultimate “valve” before membrane rupture.

Combining electrophysiological analyses of *Ec*MscS mutants with modeling, Rowe et al. (2014) provided an exciting new perspective on MscS function. The authors addressed the question of macromolecular crowding (Ellis, 2001), which reduces the intracellular volume of solvent available for other molecules, upon MscS functioning. They show that besides its role as an eﬄux valve, MscS is also one of the sensors of internal crowding of large-molecular-weight compounds. They argue for a function of MscS in turgid walled-cells on the maintenance of their volume, shape, and mechanical strength by avoiding excessive draining. Considering the thinness of the cytosol compartment (2–10% of the plant cell volume), which is probably highly crowded, this provides an interesting new function to look for in the MscS-like channels of plant plasma membranes.

### TREK-1: A POLYMODAL CHANNEL

Mechanosensitive channels can be modulated by numerous stimuli other than mechanical ones and TREK channels which are well described in mammals constitute a good example. TREK means TWIK-related potassium channel, TWIK standing for “Tandem of two-pore K^+^ domains in a weak inwardly rectifying K^+^.^′′^ In mammals, TREK belongs to the two-pore domain potassium channel (K_2p_) family and, as a potassium channel, is responsible for cell repolarization, thus controlling both the resting and the dynamic electrical activity of cells. TREK-1, TREK-2, and TRAAK are the only members being mechanogated and TRAAK is the only eukaryotic MS channel for which crystal structures have been determined (Brohawn et al., 2012, 2013), with the bacterial MscS and MscL being the only other MS channels crystallized so far. TREK-1 together with TRAAK, as bacterial MS channels, retain their mechanosensitivity when reconstituted into liposomes (Brohawn et al., 2014) indicating that they are sensitive to stretch without the need for a second messenger or any form of tethering from the cytoskeleton or the extracellular matrix. This common behavior of MscL, MscS, TREK-1, TRAAK shows that the force from lipid (FFL) principle, first proposed for *E. coli* MS channels of spheroplast by Martinac et al. (1987), can be generalized to structurally unrelated eukaryotic channels. The FFL is a fundamental physicochemical principle based on the fact that the self-assembled bilayer necessitates inherent forces that are large and anisotropic. Then, proteins embedded in the bilayer are subjected to these push and pull forces. The principle of FFL and its relevance to MS channels in biophysical and physiological contexts was recently illustrated by Teng et al. (2014).

Several structure function studies also provided crucial information for a better understanding of the gating of these channels and together these approaches contribute to make TREK, one of the most studied MS channels with the bacterial MscS and MscL. Apart from the bilayer itself, TREK-1 activity is regulated by a plethora of stimuli (**Table 1**). Its activity is up-modulated by heat, intracellular acidosis, depolarization, volatile anesthetics and down-modulated by extracellular activation of PKA and PKC phosphorylation pathways. In addition, stimulation of Gq-coupled receptors, including metabotropic mGluR1, and mGluR5 receptors, inhibits TREK-1 activity (see Dedman et al., 2009; Noel et al., 2011 for review). More directly related to mechanical stimulation, TREK-1 is modulated by heat, lipids (lysophospholipids and polyunsaturated fatty acids) and also by the cytoskeleton acting as a tonic repressor (Lauritzen et al., 2005; Peyronnet et al., 2012). Aside from these regulations, TREK channels are characterized by the existence of several variants produced by alternative splicing and alternative translation initiation also contributing to the diversity of TREK functions. To date, no TREK analog has been found in sequenced plant genomes.

In mammals (humans/mice) TREK-1 has a wide tissue distribution and with its complex gating regulation is involved in diverse biological processes. It plays a central role in ischaemic and epileptic neuroprotection, vasodilatation, depression (Heurteaux et al., 2006), general anesthesia (Heurteaux et al., 2004) and pain perception (Alloui et al., 2006).

### PIEZO: A LARGE CHANNEL WITH A ROLE IN MECHANOPERCEPTION

Piezo protein (from the Greek “p*ί*esi” meaning pressure) discovered by Coste et al. (2010) was shown to be an essential component of a cationic non-selective MS channel from mouse neuroblastoma cells (**Table 1**). The protein is approximately 2500 amino acids long with 24–36 predicted trans-membrane domains showing no homology to other already known MS or voltage sensitive channels. In the membrane, Piezo1 proteins are found to be organized in a gigantic homotetrameric structure but the experiment did not show that the pore-forming unit was a tetramer.

Coste et al. also cloned a homologous gene called Piezo2 from mouse dorsal root ganglion cells with similar electrophysiological properties. After expression, purification, and reconstitution in artificial lipid bilayers, Piezo1 was shown to be the pore-forming subunit (Coste et al., 2012). Piezo1 mutations were associated with autosomal dominant hemolytic anemia (Zarychanski et al., 2012). Patapoutian’s group also demonstrated that Piezo2 is expressed in a mechanoreceptor complex in mouse skin and is required for gentle touch perception (Woo et al., 2014). In the same way, Piezo proteins in *Drosophila* larvae have been shown to be crucial for responses to noxious mechanical stimuli (Coste et al., 2012).

Piezos are fascinating proteins (see Gottlieb and Sachs, 2012; Nilius and Honore, 2012 for reviews) and it is still unclear why these proteins are so big. It is tempting to hypothesize that the large number of transmembrane domains along with the proteins’ large size could constitute an effective sensor for membrane curvature. In any case, this unusual structure for a mechanotransducer is likely to suggest other functions. Piezo, an evolutionarily conserved protein, presents a single homolog in the genome of the model plant *Arabidopsis*, providing an interesting new candidate for plant mechanosensors.

## ACCESSING MS ACTIVITY AT CELLULAR LEVEL

### PATCH-CLAMP COMBINED WITH HIGH SPEED PRESSURE-CLAMP REVEALS INTIMATE PROPERTIES OF MECHANOSENSITIVE CHANNELS

**FIGURE 2:**
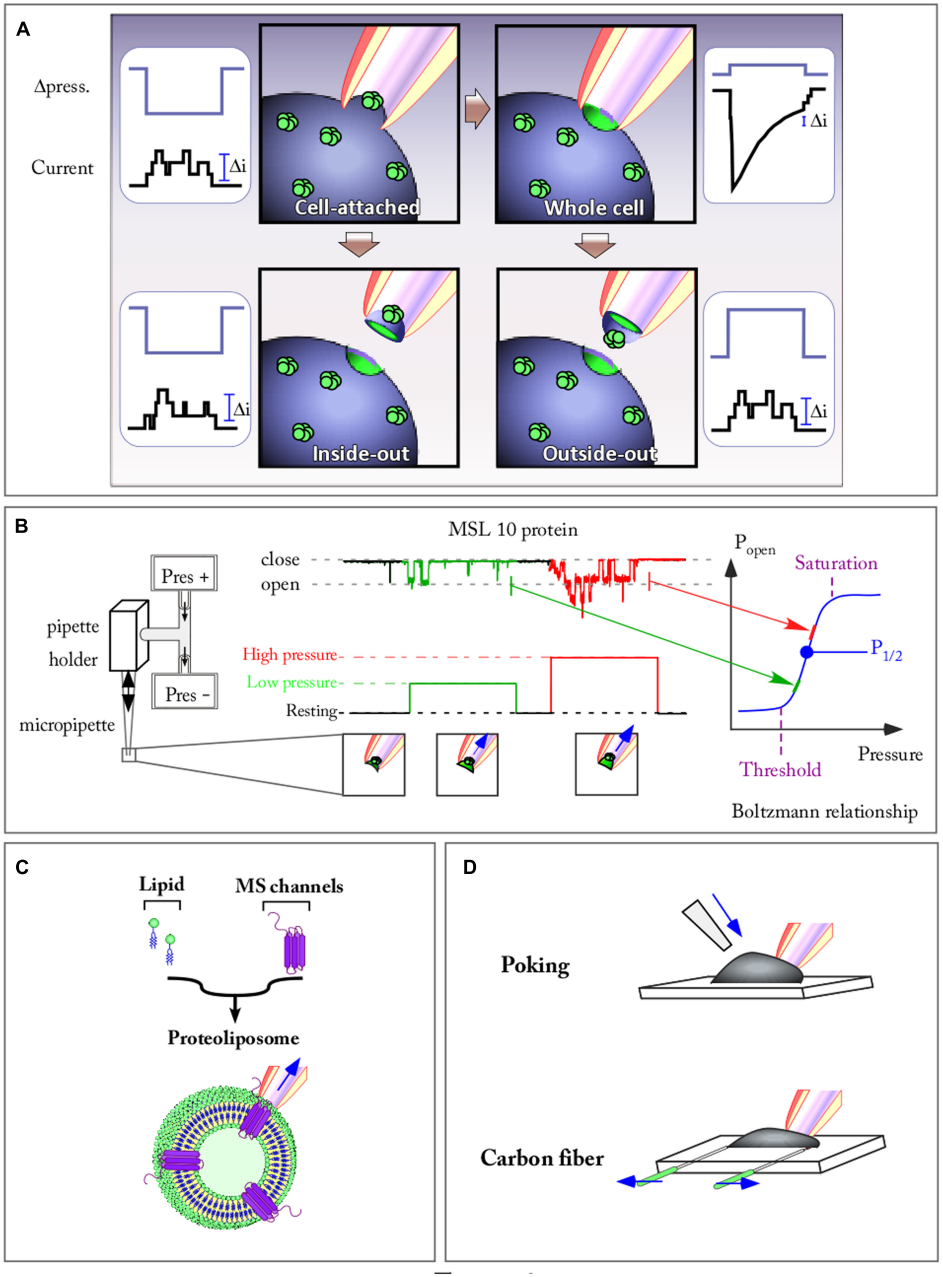
**Patch clamp combined with fast speed pressure stimulation allows to study kinetic properties of mechanosensitive (MS) channels. (A)** The four patch clamp configuration allowing to record either single channel current (Cell attached, Inside-out, and Outside-out) or a population channel current (Whole cell) are represented. Channels are stimulated by applying a pulse of pressure against the membrane. **(B)** Recording of the *Arabidopsis* MSL10 channel activity in outside-out configuration. The activity of the channel is elicited by pulses of increasing pressure. The open probability (P_open_)-pressure relationship of the channel fit a Boltzmann function on which are mentioned: the activation threshold, the half pressure activation (P_1/2_), the pressure of maximum activation. **(C)** MS channel reconstituted in a spherical proteoliposome. **(D)** Poking and carbon fiber techniques allow to apply membrane stretch while recording the cell (arrows show forces applied to the system).

Patch clamp is a powerful technique that allows the recording of channel activity with a high resolution in terms of time (ms) as well as ion flux (pA). **Figure 2A** is a summary of the different configurations that can be achieved in patch clamp. The use of whole cell configuration allows the recording of channel population activity present on a membrane while the other possible patch configurations select a small number of channels at the tip of the pipette, enabling resolution of single channel activity. These latter configurations are reached through a cell attached configuration that maintains intracellular integrity, thus complying with the transduction pathways, or through an excised patch allowing a better characterization through which the ionic environment on both sides of the membrane is fully controlled. Excised patch configurations also allow us to test the role of the cytoskeleton. It has been shown that cytoskeleton elements are strongly destabilized in this configuration in comparison with cell attached mode (Lauritzen et al., 2005; Peyronnet et al., 2012). Only excised and cell attached patches allow application of a large range of pressures and therefore, the highlighting of the relationship between open probability and membrane tension of a single channel. The development of the pressure clamp system in the nineties (McBride and Hamill, 1992; Hurwitz and Segal, 2001) has become a key tool for applying fast pressure steps to membrane patches (**Figure 2B**). The ability to measure channel relaxations following step changes in positive/negative pressure in combination with patch clamp techniques has launched many studies on the analysis of the time, voltage and pressure dependence of the opening and closing of MS channels from different organisms, exemplified in **Figure 2B** with the *Arabidopsis* mechanosensitve MSL10 channel. Thus the relationship between open probability/pressure fits a sigmoid curve called a Boltzmann function, indicating the threshold and maximum (saturation) tension values of the channel. The slope of the sigmoid depicts the strength of the channel dependence toward membrane tension. The most artificial, but best controlled situation, is encountered when the MS channel is reconstituted in an artificial membrane bilayer of a spherical shaped proteoliposome (**Figure 2C**). Here, not only is the ionic environment well controlled, but the lipid composition of the membrane is also mastered. The absence of cytoskeleton elements is also a strong advantage allowing direct access to membrane mechanical properties without the cytoskeleton interfering with either the channel or the membrane itself. In these different configurations, applying a positive or negative pressure will allow delivery of controlled tension to the membrane if curvature of the patch is measured (Suchyna et al., 2004). Following this, single MS channels can be characterized with a very good time resolution (milliseconds) either in native or artificial membranes.

Careful and detailed analysis of physical phenomena that are generated on the patch of membrane sealed at the pipette tip have led authors to underline the limit of the patch-pressure clamp technique. Although the pressure in the pipette can be controlled precisely, its conversion into local tension, the parameter that activates MS channels, is not straightforward. In his paper, Sachs (2010) discusses the different components involved in the generation of local mechanical stress: far-field tension, phase separation, the cytoskeleton, and the adhesion energy between the membrane and the patch pipette. The pressure-clamp technique is currently one of the easiest ways to mechanically activate and record single channels, but various methods of mechanical stimulation have been developed to stimulate and record channels at the whole cell level.

### OTHER TECHNIQUES TO ACTIVATE MS CHANNELS

The advantages and limitations of these techniques are a matter of active discussion (Kamkin and Kiseleva, 2008). In **Figure 2D** two of these techniques are illustrated. The poking technique is commonly used to apply mechanical stimulation to single cells. While recording in whole cell configuration, the stimulation is generally achieved using a fire-polished glass pipette (tip diameter 3–5 μm). Controlled downward movements of this probe press the cell against its support, thus activating MS channels. This technique notably led to the discovery of Piezo channels (Coste et al., 2010).

The carbon fiber technique allows a controlled axial stretch of the cell and was first developed on cardiomyocytes (Le Guennec et al., 1990). Carbon fibers are attached to the cell membrane *via* electrostatic forces (the same forces that seal the patch-clamp pipette to the membrane). Carbon fiber bending is converted into forces generated by the cell (such as in response to an osmotic shock) while a microelectrode records the current flowing through MS channels (**Figure 2D**). Instead of using carbon fiber, it is also possible to use glass capillaries coated with glue. These capillaries are attached to a force transducer allowing a direct recording of the force generated by the cell while stretching. These techniques are also used to test mechanical properties of tissues such as stiffness. Regarding the poking technique, the amplitude of the downward movement compared to the cell diameter might, in some experiments, produce excessive deformation. This prompts a use of this technique with stimuli generating cell strain physiologically relevant. Until now these techniques have exclusively been developed on animal cells. Their adaptation to plant cells is of major interest in order to develop our knowledge of plant MS channels.

Both in-plane membrane tension and membrane curvature have been shown to activate MS channels. This can be achieved by asymmetric incorporation of cone-shaped amphipaths (Martinac et al., 1990). These molecules, able to insert selectively in one membrane leaflet (Sheetz and Singer, 1974), create positive or negative curvature. The activation by amphipaths differs from in-plane membrane tension because it implies local membrane curvature as the activation factor (**Table 1**; Yoo and Cui, 2009). MscL and MscS are activated, even in the absence of applied pressure, when cone-shaped lysophosphatidylcholine is inserted into the membrane (Vasquez et al., 2008; Mukherjee et al., 2014). This activation property is also shared by the eukaryotic channel. TREK-1 for example, is opened by crenators, while it is closed by cup-formers. (Patel et al., 1998; Maingret et al., 2000).

## MS CHANNELS ARE FAST TRANSDUCERS OF MEMBRANE TENSION CHANGES

**FIGURE 3:**
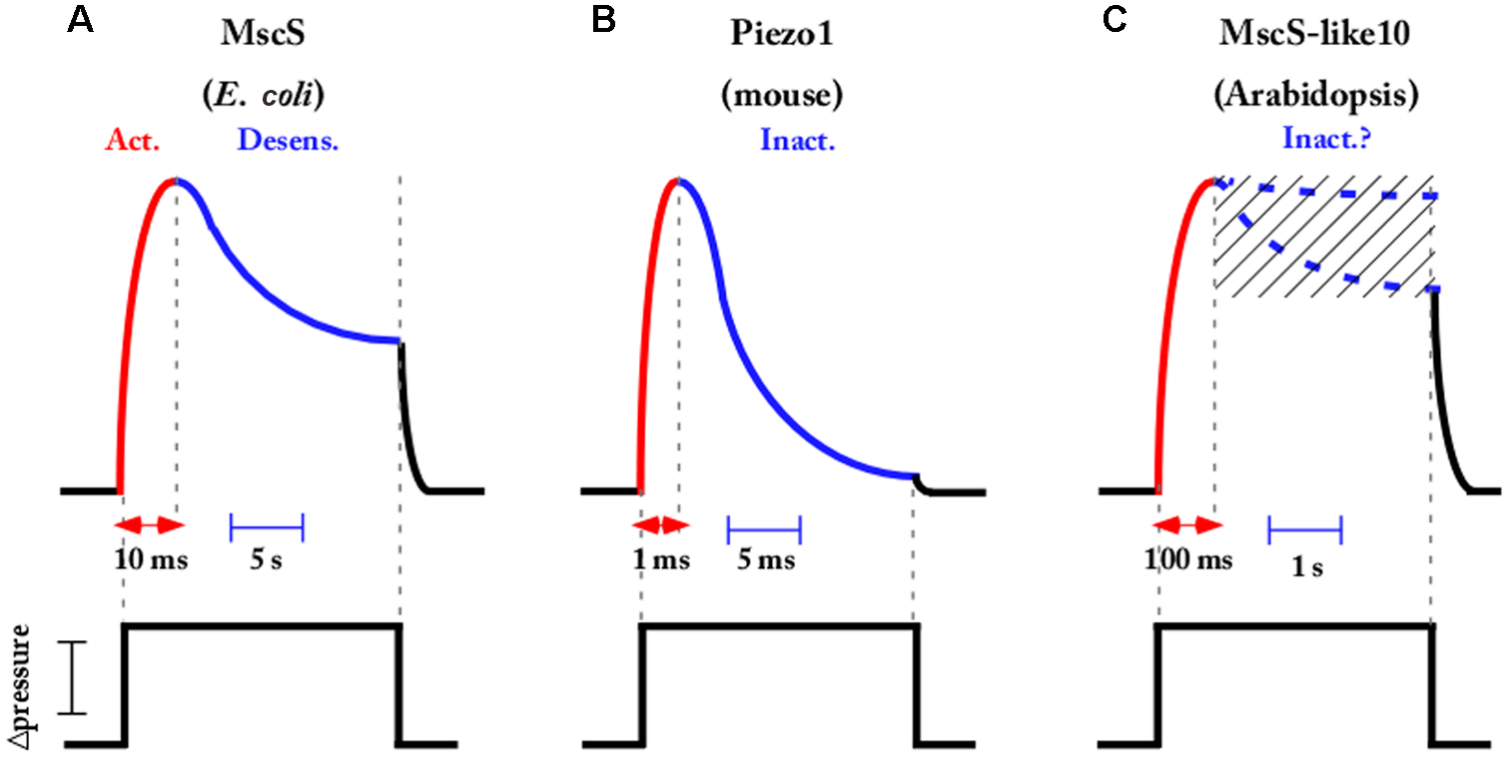
**Most MS channels exhibit fast activation followed by a slower inactivation or desensitization kinetics in response to pressure stimulation.** Schematic representations showing that activation inactivation and desensitization kinetics occurred in the time range of ms to s depending on the channel **(A)** *Escherichia coli* MscS [inspired from Boer et al. (2011)], **(B)** Piezo mouse channels [inspired from Coste et al. (2012)], and **(C)** *Arabidopsis* MscS-like 10 channel [inspired from Maksaev and Haswell (2012)]. For MscS-like 10, the presence or absence of inactivation not being definitively settled, the two possibilities are mentioned. Channels are elicited by a step of sub-saturating pressure applied to the membrane.

The ability to precisely control mechanical stimulation with the fast pressure clamp system has provided valuable kinetics information on the activation, inactivation, and deactivation of MS channels from different organisms. The MscS, acting as a tension-driven osmolyte release valve in bacteria, exhibits rapid activation in the 10 ms time range (Boer et al., 2011). In response to sustained and moderate stimulus *Ec*MscS exhibits complex desensitization kinetics that is composite of both channel adaptation that is likely linked to membrane mechanics and inactivation of the channel (Belyy et al., 2010; Boer et al., 2011; Kamaraju et al., 2011; Cox et al., 2014). In **Figure 3A**, the current decay of the MscS population is represented in the case of a sub-saturating tension applied to the membrane. In such conditions, the fraction of decrease due to inactivation is dominant over the fraction due to adaptation. However, using a specific pressure protocol (not presented in **Figure 3**), combining prolonged conditioning steps interspersed with short saturating pulses (Akitake et al., 2007; Kamaraju et al., 2011) allowed for distinction between these two interrelated processes and led Rowe et al. to show that inactivation is increased in the presence of crowding agents (Rowe et al., 2014). The potential physiological relevance proposed as a result of this channel adaptation and this inactivation crowded dependent is to link sensitivity to both membrane tension and crowding pressure in order to limit the dissipation of the vital gradient and to maintain cell strength and turgor during hypoosmotic shock.

Several eukaryotic MS channels also exhibit complex kinetics, with TREK and Piezo being well-known examples. They both activate in a very short period of time, in the range of a few milliseconds, and then inactivate under sustained membrane tension (**Figure 3B**). TREK desensitization was reported as weakly or not at all dependent on the cytoskeleton and the voltage in mammalian cells (Honore et al., 2006) and this was recently confirmed in artificial lipid bilayers where TREK and TRAAK were still inactivated (Brohawn et al., 2014). Piezo1 inactivation is abolished when Zn^2+^ instead of Mg^2+^ is perfused on the intracellular face and no other regulators have been described until now (Gottlieb et al., 2012). Mechanisms modulating desensitization of MS channels are far from being fully understood and deserve further study as they constitute a major characteristic of ion channel activity.

From a functional perspective, inactivation can have different roles. One role of inactivation could be to protect the cell against nonspecific responses. Indeed, inactivation guarantees that if the channel should open it cannot stay open for a long period of time. Inactivation also means that channels become desensitized to the stimulation, so if the stimulation occurs at a high frequency, the first stimulus will activate channels but not the following stimuli, and in this case the channel can act as a frequency filter.

Concerning MS channels in plants, very little electrophysiological data has been available to date. The recent discovery of anion permeable MscS-like and cation permeable MCA channels in *Arabidopsis* has not yet given the authors the opportunity to deliver a complete kinetics characterization. However, a recent electrophysiological study of AtMscS-like10 expressed in *Xenopus* oocytes (Maksaev and Haswell, 2012) indicates slow opening and closing kinetics of the channel in response to sharp steps of tension applied to the membrane. Unlike *Ec*MscS, but like many other prokaryotic MscS-like channels (MscSP, MscCG, MscK) (Nakayama et al., 2013; Petrov et al., 2013), no inactivation of the plant homologous under sustained tension was detected (for a review see Cox et al., 2014). The authors of the present paper also found activation and deactivation kinetics, in the 0.1 s range, when overexpressing AtMscS-like10 in a homologous system (**Figure 3C**). The existence or not of an inactivation process of the plant MscS-like10 channel is still under investigation and might depend on the expression system used (*Xenopus* oocyte/plant protoplast). Such an inactivation process would mean accommodation of the cell under a sustained mechanical stimulation as occurred in *E. coli* for its MscS counterpart. Further exploration is needed in plants in order to obtain a complete picture of plant MS channel function.

## PLANT MS CHANNELS

### PLANT CELLS PROVIDE A PECULIAR “MECHANICAL ENVIRONMENT” FOR MS CHANNELS

Plant and animal cells have developed their own intracellular and extracellular matrices which differ in organization and structural composition. Plant cells exhibit very stiff pecto-cellulosic walls, notably because of the presence of cellulose microfibrils which has stiffness comparable to that of steel. Conversely, animals have wall-less cells in which the mechanical properties of the membrane are heavily dependent on the cytoskeleton network. In plants, microtubules form a dense cortical network and actin creates a slight internal network while in animal cells the situation is inverted. In the latter, cell mechanics are highly relayed to an actin contractile cytoskeleton and membrane whereas in plants, the presence of a stiff extracellular matrix is designed to moderate the contribution of the cytoskeleton. In animals, there is increasing evidence of a significant role for actin as a relay in MS channel activation (Lauritzen et al., 2005; Peyronnet et al., 2012). In plants, only one study performed on MS guard cell channels (combining electrophysiology and pharmacology) indicates channel activation when actin filaments are disrupted and channel inhibition with stabilization of actin (Zhang et al., 2007). Microtubules which represent the major mechanical component of the plant cytoskeleton (Nick, 2013) have not yet been investigated for their role in MS channel activation.

**FIGURE 4:**
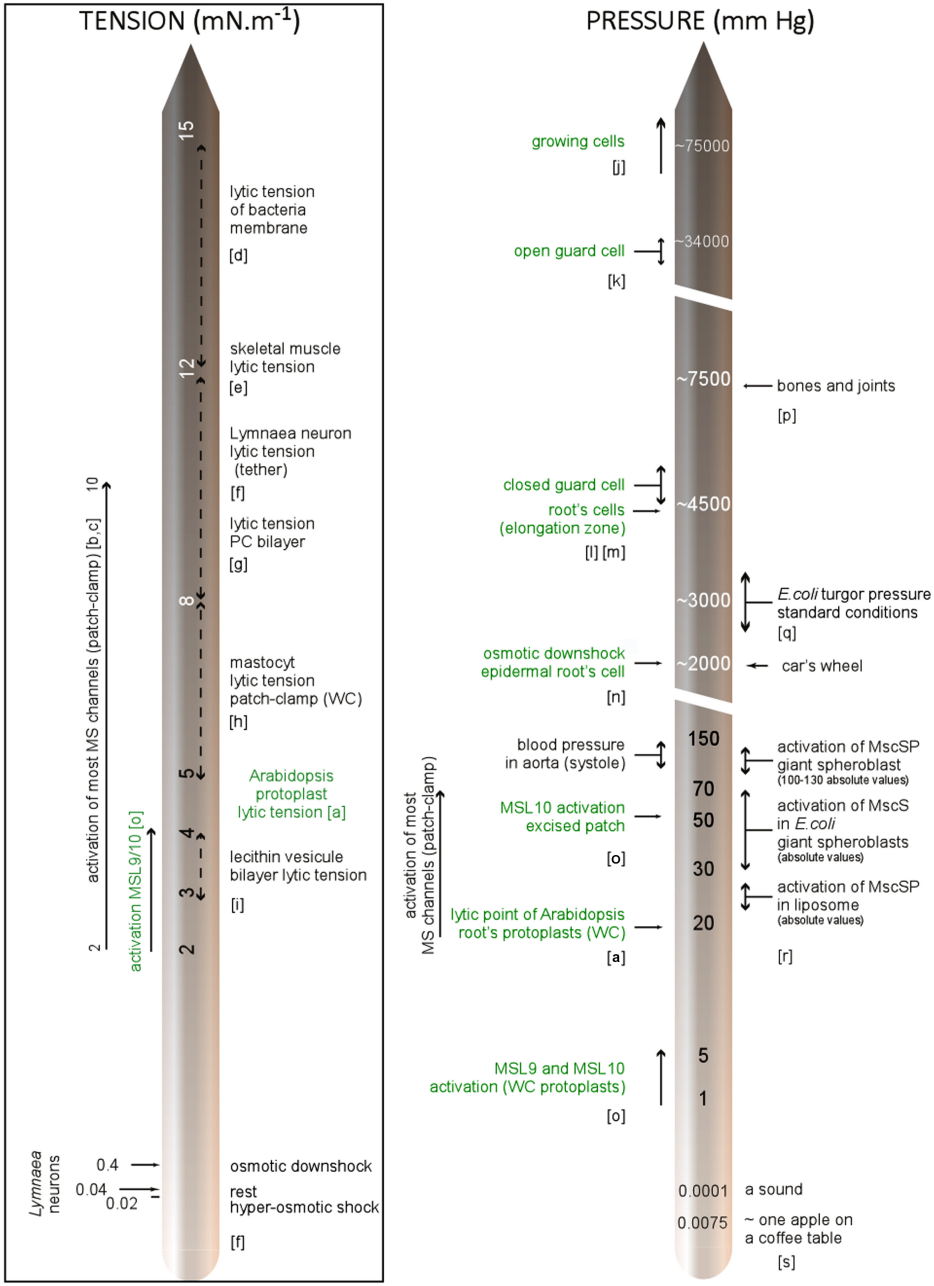
**Membrane tension and pressure within cells: milestones and experimental data.** References; a: Wolfe et al. (1985), b: Gustin et al. (1988), c: Sachs and Morris (1998), d: Boer et al. (2011), e: Nichol and Hutter (1996), f: Dai et al. (1998), g: Evans and Ludwig (2000), h: Solsona et al. (1998), i: Kwok and Evans (1981), j: Meckel et al. (2004), k: Cosgrove (1996), l: Franks et al. (2001), m: Pritchard et al. (1990), n: Shabala and Lew (2002), o: Haswell et al. (2008), p: Sukharev and Sachs (2012), q: ( Wood, 1999), r: Petrov et al. (2013), s: Morris and Homann (2001). In green: characteristics related to plant, WC, whole-cell configuration; PC, phosphatidylcholine.

Looking deeper into the structure of the plant wall-membrane-cytoskeleton continuum, it is worth considering the contact points connecting these compartments. For the membrane-cytoskeleton, this is exemplified by a plethora of microtubule-associated proteins identified by the recently sequenced genomes of model plants (Gardiner, 2013). Cytoplasmic linker associated protein (CLASP) represents one example of these recently identified anchorage proteins although the intimate mechanism still needs to be investigated (Brandizzi and Wasteneys, 2013). The abnormal root cell swelling and increased microtubule ordering of *clasp-1* mutants (Ambrose et al., 2007) stresses the importance of this linkage-protein in cell shaping and suggests a role in force cortex sensing. Another indication of the link between cell wall and microtubule networks is the parallel patterning of cellulose microfibrils with microtubules (Szymanski and Cosgrove, 2009). Furthermore, live cell imaging experiments in hypocotyl provide evidence that the cortical cytoskeleton guides the movement of cellulose synthase complexes (Paredez et al., 2006; Chan et al., 2011). The physical and biophysical significances of these tight contacts in terms of stress information is well discussed by Szymanski and Cosgrove (2009) and Cosgrove and Jarvis (2012) but little investigated by the scientific community. In addition to the contribution of their structural elements, the mechanical properties of cells largely depend on osmotic pressure commonly in the range of 2,300–6,800 mm Hg (0.3–0.9 MPa) in growing cells (Cosgrove, 1993) and estimated at up to 75,000–375,000 mm Hg (10–50 MPa) for wall tensile stresses. To bring these values into context and help realize how peculiar the mechanical environment is in plants, we have illustrated some pressures recorded in plant cells with regard to some milestones and pressures used in electrophysiology to activate MS channels. As tension rather than pressure activates MS channels, we have also illustrated tensions even if very little data has been available in the literature until now (**Figure 4**).

While turgor pressure is uniform and isotropic within the cell, wall stresses are not generally uniform, but depend on cell geometry, cell wall thickness, and wall mechanical properties. In the case of a growing cell (often encountered in plants) physical properties are dynamic, with wall extensibility varying within minutes or even seconds (Wolf et al., 2012).

All the points mentioned require integration into a biophysical vision of the plant cell. Combining molecular physics and modeling approaches will lead to drawing a map of forces within the cell, allowing crucial questions to be answered such as; what is the mechanical contribution of the cell wall? Does it absorb most of the tension applied to the cell or does the plasma membrane beneath behave as the element under tension? Are the linkage points hot spots for stress? Then, MS channels characterized by the patch clamp technique (in simplified environments: bilayer, proteoliposome, protoplast) could be mapped with the cellular strain distribution in order to understand where and when they are physiologically relevant.

### PROBING MECHANICAL FORCES IN PLANT. A GREAT JOB TO DO IN THE NEAR FUTURE!

A crucial step in understanding the role of mechanosensors is to know where and when mechanical forces occur within cells and tissues and to be able to quantify them. Membrane curvature as well as membrane and cytoskeleton tension, which are essential MS channel modulators, have been poorly described until now. The main reason is that until recently, there were no techniques to study these forces *in vivo*. Fortunately, today this important area controlling many biological processes is regaining a lot of interest due to the emergence of new tools.

It has become better and better established that the cytoskeleton is a powerful regulator of membrane tension, shape and organization in animal cells (Ofer et al., 2011; Saravanan et al., 2013; Al-Rekabi et al., 2014). In plant cells, for which its role is less established, probing its mechanical state will be very helpful to better understand its effect on MS channels. Fluorescent probes based on the Förster resonance energy transfer (FRET) technique were recently engineered to sense tension variations within the cytoskeleton (Meng and Sachs, 2011; Guo et al., 2013) with single piconewton sensitivity (Grashoff et al., 2010). Adaptation of these sensors to plant cells represents an exciting challenge and will allow mechanical forces within the plant body to be mapped.

To complete this “strain mapping” *in planta*, the use of fluorescent probes sensitive to curvature (asymmetry in lipid packing) will enable the drawing of the membrane micro curvature map, another important MS channel regulator. Genetically encoded fluorescent probes already exist in animals like the BAR domain proteins (Bin1-Amphiphysin-Rvs167; Peter et al., 2004; Ren et al., 2006) which have homolog in plants (Zhuang and Jiang, 2014) and α-Synuclein (Pranke et al., 2011). Interestingly, these curvature sensors have been shown to be efficient in different organisms (Pranke et al., 2011), making the translation to plants realistic.

Promising probes for sensing changes in the membrane mechanical state are MS channels themselves. Very recently, Wang et al. (Wang et al., 2014) revealed MscL’s conformational changes using single molecule FRET. Using engineered MS channels with a FRET sensor and a known activation threshold allowed direct probing of mechanical changes. Other techniques with non-genetic probes are also being developed. For example, based on oil droplet deformations, Campás et al. (2014) describe mechanical forces within living embryonic tissues. Deformable microchips also exist to monitor tissue deformations (Kim et al., 2012). Bio-imaging approaches combining the use of such tension/curvature probes with the subcellular localization of pre-labeled (with fluorescent protein) MS channels will allow the correlation of tension distribution with the presence of the channel. Such probes would help further understanding of whether MS channels are preferentially localized in the area of a curved membrane, close to cytoskeleton structures, or areas of membrane under tension, leading to the elucidation of the regulation and the role of MS channels *in vivo*.

### GENETIC APPROACH

Several genetic strategies exist to highlight the roles of MS channels at the whole organism level. KO mutants have been extensively used to reveal MS channel functions. Bacterial Msc S and L channels (Levina et al., 1999), mammalian TREK-1 (Alloui et al., 2006; Heurteaux et al., 2006), and Piezos (Kim et al., 2012; Woo et al., 2014) are some successful examples illustrating this strategy. For plant MS channels the KO mutant approach is only in its infancy. Referring to TREK and TRAAK MS channels candidates mentioned in the first section of this review, no homologs of these mammalian proteins were found in currently sequenced plant genomes. By contrast, Piezo, an evolutionarily conserved protein, is expressed in invertebrates, plants, and protozoa. In plants, Piezo has only one homolog making this putative plant mechanosensor even more interesting, which urges experimenters to apply the KO strategy for this gene. In the same way, considering the prokaryotic MscS channels, homolog are present in plants but not in mammals. AtMSL 2 and 3, the closest *Arabidopsis* member of *E. coli*, were shown by Haswell and Meyerowitz (2006) and Wilson et al. (2011) to be involved in chloroplast shaping and Z-ring elaboration. Although AtMSL9 and AtMSL10 were proved to be channels activated by membrane stretching in *Arabidopsis* (Haswell et al., 2008; Peyronnet et al., 2008; Maksaev and Haswell, 2012) the KO strategy applied to these genes and related AtMSL4, AtMSL5, AtMSL6 members did not reveal phenotypes. A complementary approach to develop in this situation is the generation of gain of function (GOF) mutants. Indeed, MS channels must be closed in resting conditions in order to avoid unsuitable elicitation and unnecessary ion gradient dissipation. Hence, finding the “window of activation” is mandatory in order to obtain a phenotype. The use of conditional GOF, either in channels constitutively open or with a lowered activation threshold, can considerably enlarge this window and help reveal functions. This strategy was applied notably for Piezo2 and might be insightful for MSL in plants.

### COUPLING CHANNEL ACTIVATION TO SECONDARY MESSENGERS?

The immediacy of the MS channel activation and the earliness of cellular ion fluxes and ROS variations (Monshausen et al., 2009; Coutand, 2010) in response to membrane stretching raises the question of the nature of the coupling between the channel and secondary messengers. Two types of situations might be considered. First, the simplest is due to the high capacity of the MS channel to eﬄux ions. As a consequence, an activation of the channel will lead to a loss of osmolyte occurring in a few minutes. This situation will lead to rapid protection of the cell against damage from hypoosmotic shock, as it does in *E. coli* upon activation of MscS and MscL channels. In the latter case, no immediate coupling with a second messenger is required since the predominant effect is a massive eﬄux of ions passively moving down the electrochemical gradient. Secondly, in less severe situations, the way in which the coupling occurs between MS channel activation and early Ca, pH, and ROS variation is still unknown. In response to either mechanical curvature of the plant root (Monshausen and Gilroy, 2009) or the oscillatory cell wall deformation occurring during hypocotyl cell elongation (Wolf et al., 2012), the earliest response is a rise in cytosolic calcium. In their attempt to connect plasma membrane stretching with Ca^2+^ influx, authors almost always, speculate about the intervention of a MS Ca channel. Until now, the only proposed MS channel suspected to be Ca permeable has been MCA1, which generates cation currents upon membrane stretching when expressed in *Xenopus* oocytes (Furuichi et al., 2012). Then, calcium influx would be amplified by a Ca-induced Ca-released mechanism requiring intracellular calcium stores. Another way to effectively link MS channels with Ca variation would be through an electrical coupling. Indeed, voltage dependent channels selective for calcium have been described on the plasma membrane (Miedema et al., 2008). These channels are activated by plasma membrane depolarization. Now, let us consider a membrane in which a MS AtMSL10 channel and a calcium voltage dependent channel coexist. Stretching this membrane will activate the AtMSL10 anion permeable channel (Haswell et al., 2008; Maksaev and Haswell, 2012) which will bring the membrane potential toward an equilibrium potential for anions (Cl^-^ and NO_3_^-^), meaning a large depolarization. In turn, a Ca voltage dependent channel will be activated by electrocoupling (Action Potential is an example of electrocoupling between Na^+^ and K^+^ channels in animals) producing an inward Ca current. In order to check this scenario it is of major interest to compare, in the near future, the Ca signature of mechanically stimulated wild type versus KO plants for MS genes such as MSL, MCA, or Piezo.

### THE PECULIAR STATUS OF TONOPLAST MS CHANNELS

Over the past 20 years, distinct MS channel activities have been characterized using the patch clamp method (for review see Haswell, 2007). The majority of them were described on the plasma membrane which was easily amenable to the patch clamp technique. The few studies performed on mechanogated channels on plant tonoplast did not allow for molecular identification. However, it is in 2003, in yeast (Zhou et al., 2003), that the first vacuolar membrane MS channel belonging to the TRP family was characterized. Channels of the TRP superfamily are associated with sensations of force, temperature, and chemicals in animals. These channels are formed of tetramer subunits selective for small cations. To date, no homologous has been found in plant (Kung et al., 2010; Eijkelkamp et al., 2013). The yeast TRP1, like other TRP, is polymodal, responding to voltage, membrane stretch force, and cytosolic Ca^2+^ concentration. In plant, only three studies (Alexandre and Lassalles, 1991; Badot et al., 1992; Maathuis, 2011) report the presence of mechanogated channels in the tonoplast membrane. The most meticulous characterization concerns a beetroot (*Beta vulgaris)* MS non-selective channel activated by hydrostatic and osmotic pressure but inhibited by the MS channel blocker gadolinium (Alexandre and Lassalles, 1991). It was recently shown by Maathuis (2011) that vacuolar channels from the two pores K^+^ family (TPKs) from the three species *Arabidopsis*, rice, and barley were stretch sensitive. The dependence of these channels on membrane stretching and osmotic gradients led the author to propose a role for intracellular osmosensors for TPKs contributing to K^+^-released induced by hypo-osmotic shock. Considering the large size of the vacuole (the largest cell compartment being up to 90% of the cell volume) and the presence of MS activities in its membrane, it remains crucial to further study tonoplast mechanosensitivity. It is noteworthy that the physical forces applied to this membrane and its links with the cytoskeleton should be rather distinct to those of the plasma membrane. In conclusion, the molecular identification of tonoplast MS channels and the understanding of the role of the vacuole in mechanoperception are part of the next exciting challenges.

## OUTLOOK

Despite many structural and mechanical differences, MS channels were conserved in plant and animal cells with similar characteristics. Considering the great difference in their life style, animal channels are presumably devoted to different functions to those of their plant counterparts. Therefore, it is of interest to predict in which function plant MS channels might be involved in order to design experimental conditions to reveal their role. If the ancestral role of the bacterial MscS channel is conserved, some MS channels might be involved in osmoregulation. Thus, looking at the kinetics of either cell swelling (root hair cell) or root ionic flux under different osmotic conditions will give an indication of this function. The root network also has to face obstacles and adapt to the hardness of the substrate during its anchorage role. Considering that MSLs are well expressed in root tissues (Haswell et al., 2008; Peyronnet et al., 2008), it is of interest to develop experimental devices in order to thoroughly study the root growth in relation to MS channel activity. Movement, although less spectacular than in animals, is ubiquitous in plants. It might be rapid, such as the closure of the Venus fly trap or leaflets of *Mimosa pudica,* or slow as occurs in flowers and leaves during nastic movements. All plants are also able to sense both gravity and their own shape in order to grow straight. In animals this property is called proprioception (Bastien et al., 2013). All of these reorientation movements involve osmotic pressure as well as cell curvature and are likely to require MS channels. Designing screens based on imaging to capture movement would help in deciphering the MS channel role in plants.

## Conflict of Interest Statement

The authors declare that the research was conducted in the absence of any commercial or financial relationships that could be construed as a potential conflict of interest.
